# The mir‐465 family is upregulated with age and attenuates growth hormone signaling in mouse liver

**DOI:** 10.1111/acel.12892

**Published:** 2019-01-13

**Authors:** Amy E. Elias, Bianca Kun, Ian M. C. Sabula, Gail Golomb‐Mello, Andrea Cespedes Zablah, Jill A. Kreiling

**Affiliations:** ^1^ Department of Molecular Biology, Cell Biology and Biochemistry, Brown Center on the Biology of Aging Brown University Providence Rhode Island

**Keywords:** aging, growth hormone receptor, growth hormone signaling, liver, miRNAs, mouse

## Abstract

We analyzed the small RNA transcriptome from 5‐month‐old, 24‐month‐old, and 36‐month‐old mouse liver and found 56 miRNAs that changed their expression profile with age. Among these is a cluster of 18 miRNAs that are upregulated between 50‐ and 1,000‐fold at 24 and 36 months of age. This cluster is located in a 60‐kb region of the X‐chromosome that is devoid of other coding sequences and is part of a lamin‐associated domain. Potential targets of the miRNAs in the cluster suggest they may regulate several pathways altered in aging, including the PI3K‐Akt pathway. Total transcriptome analyses indicate that expression of several potential genes in the PI3K‐Akt pathway that may be targeted by the mir‐465 family (mmu‐mir‐465a, mmu‐mir‐465b, and mmu‐mir‐465c) is downregulated with age. Transfection of the liver cell line AML12 with mir‐465 family members leads to a reduction of three of these potential targets at the mRNA level: a 40% reduction of the growth hormone receptor (GHR), and a 25% reduction in Kitl and PPP2R3C. Further investigation of the GHR 3′UTR revealed that the mir‐465 family directly targets the GHR mRNA. Cells transfected with mir‐465 showed a reduction of JAK2 and STAT5 phosphorylation upon growth hormone (GH) stimulation, resulting in a reduction in insulin‐like growth factor 1 (IGF‐1) and IGF‐1‐binding protein 3 expression. With age, GH signaling falls and there is a reduction in circulating IGF‐1. Our data suggest that an increase in expression of the mir‐465 family with age may contribute to the reduction in the GH signaling.

## 1 INTRODUCTION

Alterations in growth hormone (GH) signaling affect healthspan and lifespan in a wide range of organisms ranging from invertebrates to humans (Bartke, List, & Kopchick, 2016; Barzilai, Huffman, Muzumdar, & Bartke, 2012). The GH signaling pathway plays a key role in metabolic regulation through activation of multiple signaling pathways including the IGF‐1 signaling pathway, the MAP kinase pathway, and the PI3K‐Akt pathway. GH signaling is thought to influence lifespan through its action on the somatotrophic axis (Bartke et al., 2016). GH is synthesized in the anterior pituitary gland and secreted into the bloodstream upon hormonal stimulation where it acts primarily on the liver through binding to the GH receptor (GHR). Activation of the GHR by GH binding leads to the phosphorylation of Janus‐family tyrosine kinase 2 (JAK2), which subsequently phosphorylates several transcription factors resulting in their activation, including the STAT5b transcription factor that stimulates insulin‐like growth factor 1 (IGF‐1) expression (Alvarez‐Nava & Lanes, 2017). IGF‐1 is released into the bloodstream as a complex with IGF‐binding protein 3 (IGFBP3) and IGF acid‐labile subunit (IGFALS) where it targets numerous cell types to regulate metabolism, growth, and development.

Many studies have looked at the effects of a lifelong, global reduction in growth hormone signaling by knocking out various components of the GH signaling pathway. Interestingly, the majority of these studies resulted in healthspan and lifespan extension (reviewed in Brown‐Borg, 2015). These results appear to be applicable to humans as centenarians have higher frequencies IGF1R gene polymorphisms that result in altered IGF‐1 signaling (Suh et al., 2008). In addition, individuals with Laron dwarfism, which results from a mutation in the growth hormone receptor gene, have decreased incidence of diabetes and cancer; however, they do not have an extended lifespan (Guevara‐Aguirre et al., 2011). Taken together, these results suggest that a reduction in GH signaling has a beneficial effect on healthspan and lifespan.

However, the role of GH signaling in determining healthspan and lifespan does not appear to be that clear‐cut. GH signaling normally declines with age and this reduction has been established as a risk factor for developing diabetes, cardiovascular disease, sarcopenia, osteoporosis, and frailty (Bartke et al., 2016; Barzilai et al., 2012), and sustained elevated GH signaling increases the risk for some cancers (Barzilai et al., 2012). Delivering GH to GH‐deficient mice during the juvenile period diminishes the lifespan‐extending effects, whereas the administration of GH during adulthood does not affect lifespan (Panici et al., 2010; Sun et al., 2017). These results suggest that there is a narrow window in preadulthood when a reduction in growth hormone signaling can influence lifespan. In addition, liver‐specific reduction in IGF‐1 in middle‐aged mice had negative consequences including liver inflammation, accelerated bone loss, oxidative stress in various tissues, and increased hepatic tumors leading to a reduction in healthspan (Gong et al., 2014). These effects are thought to be caused by an increase in circulating GH, resulting from interference with the normal negative feedback loop of IGF‐1 on GH release (List et al., 2014). Collectively, these studies suggest that GH signaling must occur in an optimized manner to promote healthy aging, and that these interactions are more complex than previously believed.

The natural decline in GH signaling with age is associated with the onset of age‐associated diseases and pathologies. Therefore, the mechanisms that result in these natural changes in GH expression during our lifespans are of significant interest but remain poorly understood. Gene expression is controlled at multiple points from regulation of transcription by epigenetic features to modulation of mRNA levels by small, noncoding RNAs. MicroRNAs (miRNAs) are a class of small, noncoding RNAs that regulate mRNA expression at the posttranscriptional level. Mature miRNAs target specific mRNAs through recognition of a sequence in the 3′UTR that is partially complementary to nucleotides 2–7 of the miRNA (the seed sequence), targeting the mRNA for degradation or inhibition of translation. Here, we report that a cluster of miRNAs located in a highly heterochromatic region of the X‐chromosome is derepressed with age in mouse liver. This cluster includes members of the mir‐465 family, which we show downregulate GHR expression. A reduction in GHR expression will lead to alterations in growth hormone signaling that ultimately could affect both healthspan and lifespan.

## 2 RESULTS

### 2.1 Deep sequence analysis of the small RNA fraction in young and old mouse liver

**Figure 1:**
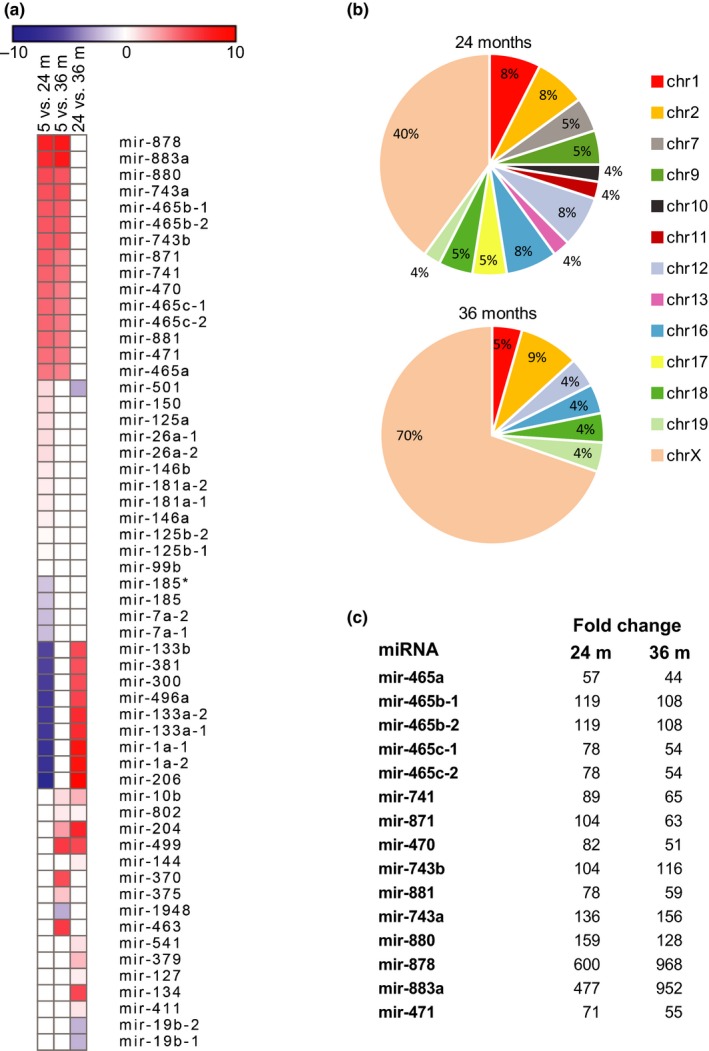
Small RNA transcriptome analysis. (a) Small RNA‐seq data from four young (5 months), four old (24 months), and four very old (36 months) male mouse livers identified 56 miRNAs that change expression levels with age (*p* ≤ 0.05, FDR ≤ 0.05). The heat map shows the level of change ranging from a decrease (blue) of log_2_ = −10 to an increase (red) of log_2_ = 10. (b) Pie charts show the chromosomal origin of the miRNAs that change expression levels with age. Note that 40% and 70% of the miRNAs that change expression levels at 24 and 36 months, respectively, are located on the X‐chromosome. (c) Fifteen miRNAs located on the X‐chromosome showed an increase in expression between 44‐ and 968‐fold in 24‐month‐old and 36‐month‐old mice (*p* ≤ 0.0002, FDR ≤ 0.002). The numbers indicate the fold increase in expression over 5‐month‐old mice. Differential expression and significance were calculated using EdgeR software (Robinson, McCarthy, & Smyth, 2010)

To investigate changes in miRNA gene expression that occur with normal aging, we performed deep sequence analysis of the small RNA fraction (<200nt) isolated from male mouse liver at different times during the mouse lifespan. C57BL/6 mice reach adulthood by 4 months of age and have a median lifespan of 27 months and a maximum lifespan of about 39.5 months (The Jackson Laboratory, 2018) Therefore, to encompass the full lifespan, we sequenced the small RNA fraction from young (5 month), old (24 month), and very old (36 month) mice. We found 56 miRNAs that changed expression levels with age (Figure 1a). The majority showed increased expression, with only 14 miRNAs decreasing in expression at 24 or 36 months of age. Further examination of the data showed that approximately 40% and 70% of the miRNAs that show significant changes in expression at 24 and 36 months of age, respectively, are located on the X‐chromosome (Figure 1b). We identified a cluster of 15 miRNAs on the X‐chromosome that are significantly upregulated at both ages (Figure 1c). Members of this cluster are expressed at very low levels in young animals and are upregulated between 50‐ and 600‐fold by 24 months of age. Their expression remains elevated (compared to 5‐month liver) at 36 months indicating that the upregulation of this miRNA cluster is not transient or reversed with advanced age.

**Figure 2:**
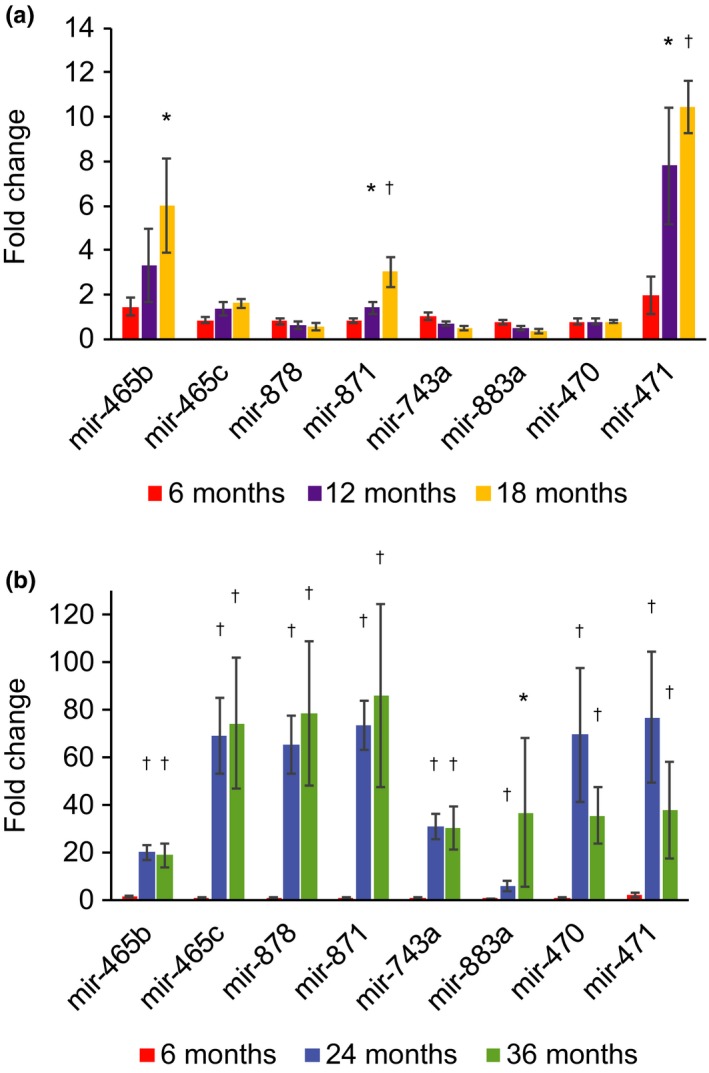
Expression of the X‐chromosomal miRNA cluster with age. Expression of eight miRNAs in the X‐chromosomal cluster was analyzed over the mouse lifespan at 6, 12, 18, 24, and 36 months of age by quantitative PCR. (a) Significant increases of 1.5‐fold and 7.8‐fold were seen in mir‐871 and mir‐471, respectively, by 12 months of age. Expression of mir‐465b increased 3.3‐fold at 12 months, but was not significant. Expression of these three miRNAs continued to increase significantly at 18 months of age, with mir‐465b increasing sixfold, mir‐871 increasing threefold, and mir‐471 increasing 10.5‐fold when compared to 6‐month‐old animals. (b) Expression of all eight miRNAs examined increased significantly at 24 and 36 months of age when compared to expression levels at 6 months of age. The increase in expression ranged from 5.8‐ to 76.9‐fold at 24 months of age and from 18.8‐ to 86.1‐fold at 36 months of age. Each bar represents the average expression level from three or four individual animals at that age performed in triplicate. Error bars represent standard errors. A representative experiment showing values for individual animals is shown in Supporting Information Figure S1. All *p*‐values were calculated using two‐tailed Student's *t* test. *p < 0.05, ^†^ *p* < 0.01 compared to 6‐month‐old animals

To determine the dynamics of this increase in expression of the miRNA cluster, we analyzed expression of the miRNAs in liver by quantitative PCR (qPCR) across the mouse lifespan. It was previously shown that these miRNAs are not expressed at significant levels outside of the testes in young mice (Landgraf et al., 2007; Ro, Park, Young, Sanders, & Yan, 2007). We found that expression of most of the miRNAs remains low through 18 months of age, confirming this previous finding. However, mir‐871 and mir‐471 show increased expression beginning at 12 months of age, and mir‐465b begins to show increased expression by 18 months of age (Figure 2a). Between 18 and 24 months of age, expression of other members of this miRNA cluster goes up significantly (Figure 2b). There was significant variation between individual animals, but overall they all showed elevated levels of expression of these miRNAs at 24 and 36 months of age (Supporting Information Figure S1).

The age‐associated upregulation of this locus was not limited to liver, as increased expression also occurred in skeletal muscle, brain, and senescent‐cultured mouse tail fibroblasts, although to a lesser extent (Supporting Information Figure S2). In skeletal muscle, expression of these miRNAs increased between fourfold and ninefold at 24 months of age, followed by a decrease to around twofold by 36 months (compared to 5 months). In brain, we did not see an increase in expression at 24 months, and only a subset of these elements showed a modest increase in expression (1.5‐fold) by 36 months of age. Senescent mouse tail fibroblasts showed between 1.7‐ and threefold induction of these miRNAs. These results suggest that the large increase in expression of these miRNAs with age is not universal and may be specific to the liver.

**Figure 3:**
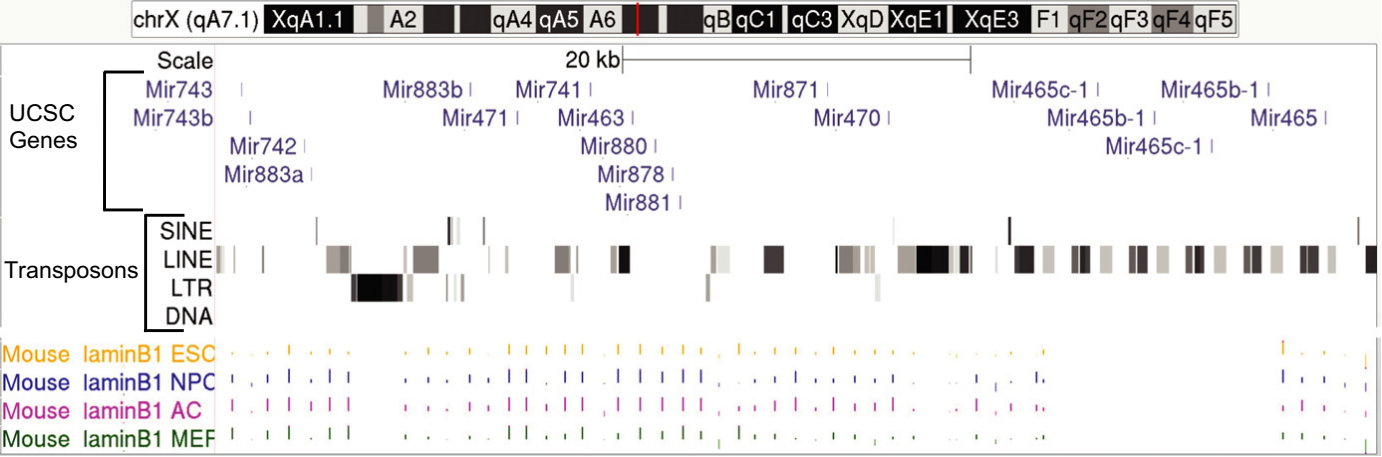
Genomic location of the X‐chromosome miRNA cluster. The cluster of miRNAs is located in a 60‐kb region of the X‐chromosome (http://genome.ucsc.edu/; Kent et al., 2002). There are a total of 18 miRNA genes located in this region, which is devoid of other coding sequences and is rich in transposons, including short interspersed nuclear elements (SINE), long interspersed nuclear elements (LINE), long terminal repeats (LTR), and DNA transposons (DNA). This region is known to be part of a lamin B1‐associated region in mouse embryonic stem cells (ESC), neural progenitor cells (NPC), astrocytes (AC), and embryonic fibroblasts (MEF; Peric‐Hupkes et al., 2010), which indicates that it is part of a heterochromatic region of the genome. The red line indicates chromosomal location of the miRNA cluster

The 15 upregulated miRNAs map to a 60‐kb region of the X‐chromosome (Figure 3). Further analysis of this region revealed a total of 18 miRNAs present in the cluster. This region is devoid of other coding genes and is rich in repetitive elements, especially in long interspersed nuclear elements (LINEs). There is an enrichment of laminB1‐binding domains in this region in other mouse tissue types, suggesting that this region is part of a lamin‐associated domain (Peric‐Hupkes et al., 2010). Lamin‐associated domains are typically comprised of heterochromatin that remains tightly repressed throughout the lifecycle of the cell. Our data showing increased expression of miRNAs in this region suggest that repression of this region may be lost with advancing age.

### 2.2 Targets of the miRNAs in the X‐cluster

**Figure 4:**
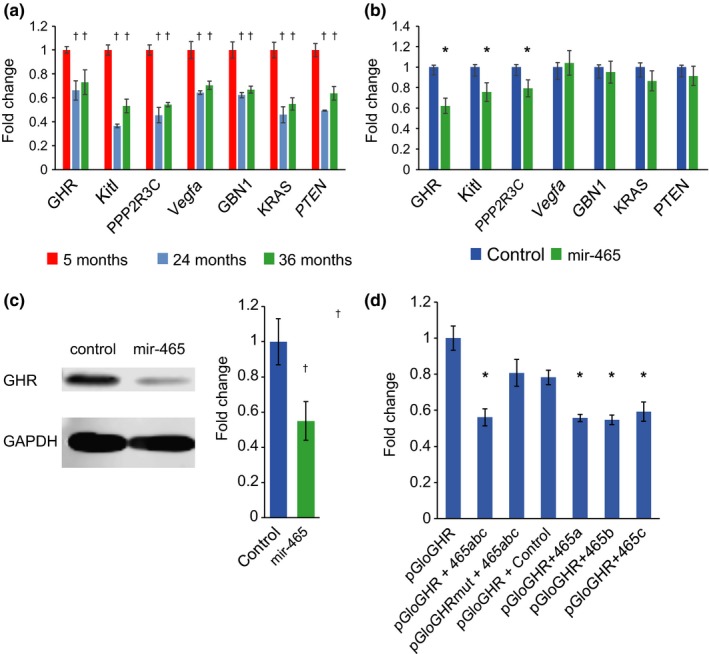
Targets of the miRNAs in the X‐chromosome cluster show reduced expression. (a) Quantitative PCR analysis of potential targets of the mir‐465 family that are reduced with age in RNA‐seq analysis verifies that expression of GHR, Kitl, PPP2R3C, Vegfa, GBN1, KRAS, and PTEN is downregulated between 27% and 64% at 24 and 36 months of age in liver. Each bar represents the average of five (24 months) or six (5 and 36 months) animals, run in triplicate. (b) GHR, Kitl, and PPP2R3C mRNA levels are significantly reduced by 38%, 25%, and 21%, respectively, in AML12 cells transfected with either pmir465abc or mir‐465c mimics when compared to control cells. mRNA expression levels of the other targets that are reduced with age in liver are not affected by mir‐465. Each bar represents the average expression value of five independent experiments. (c) Western blot analysis revealed a 50% reduction in GHR protein in AML12 cells 48 hr following transfection with pmir465abc or the mir‐465c mimic. The blot image is from a representative experiment, and the bar graph represents the average normalized intensity values of four independent experiments. (d) A 50% reduction in luminescence signal was observed in HEK‐293T cells cotransfected with a reporter plasmid containing the GHR 3′UTR (pGloGHR) along with the mir‐465 family (465abc) when compared to cells transfected with pGloGHR only. A significant reduction in luminescence signal was not seen in cells cotransfected with a reporter plasmid containing a mutated target site (pGloGHRmut) and 465abc, or in cells cotransfected with pGloGHR and a negative control miRNA. Individually, members of the mir‐465 family caused a reduction in luminescence similar to the reduction seen with the combination of all three family members. Each bar represents the average normalized luminescence value from six independent experiments. All error bars represent standard errors. All *p*‐values were calculated using two‐tailed Student's *t* test. *p < 0.05, ^†^ *p* < 0.01

The targets of the 18 miRNAs have not been well characterized in the literature. Our bioinformatic analyses of putative targets in the 3′UTR of known mRNAs show that members of this miRNA cluster have the potential to influence several major pathways known to be altered in normal aging (Table 1). Four of the top 10 pathways predicted to be affected by expression of these miRNA include the PI3K‐Akt, mTOR, MAPK, and Wnt signaling pathways. These pathways are highly intertwined and show aberrant expression in several of the major diseases of aging, including cancer. We focused our initial investigations on the PI3K‐Akt pathway since there are 69 mRNAs in this pathway that contain potential target sequences in their 3’UTR that may be recognized by at least one of 14 miRNAs in the cluster. Analysis of the liver transcriptome by RNA‐seq revealed that 28 of the 64 predicted mRNA targets showed decreased expression levels in old (24 months) and very old (36 months) mouse liver when compared to young (5 months) mice. These targets included receptors, ligands, and transcription factors associated with PI3K‐AKT signaling, indicating that attenuation of signaling by these miRNAs may occur at multiple points along the pathway. To verify that these mRNAs are reduced with age, we performed quantitative PCR (qPCR) on 5‐month, 24‐month, and 36‐month mouse liver. We focused our investigation on the predicted mRNA targets of the mir‐465 family, since nine of the 28 predicted mRNA targets in the PI3K‐AKT pathway that are reduced with age in RNA‐seq analyses are potential targets of this miRNA family. The mir‐465 miRNA family is a subset of the upregulated X‐chromosomal cluster and consists of five copies of the mir‐465 genes in a 13‐kb region of the X‐chromosome (Figure 3). We validated that seven of the predicted targets in the PI3K‐Akt pathway have reduced expression in aged mouse liver by qPCR (Figure 4a). These include GHR, Kit ligand (Kitl), protein phosphatase 2 regulatory subunit Bʺ, gamma (PPP2R3C), vascular endothelial growth factor A (Vegfa), guanine nucleotide‐binding protein beta 1 (Gnb1), Kirsten rat sarcoma viral oncogene homolog (Kras), and the phosphatase and tensin homolog (PTEN). These results establish a connection between an age‐associated decrease in expression of members of the PI3K‐Akt pathway with an increase in expression of the mir‐465 family that has the potential to regulate their mRNA stability.

**Table 1:** Pathways predicted to be affected by miRNAs in the X‐chromosomal cluster

KEGG pathway	*p*‐Value	Genes^a^	miRNAs^b^	RNA‐seq verified^c^
**PI3K‐AKT signaling pathway**	3.02 x 10^−10^	69	14	28
**mTOR signaling pathway**	2.63 x 10^−7^	17	11	7
Axon guidance	2.93 x 10^−7^	30	11	ND
GABAergic synapse	8.26 x 10^−7^	17	7	ND
Prostate cancer	1.57 x 10^−6^	20	10	ND
**MAPK signaling pathway**	6.77 x 10^−6^	45	11	9
Dopaminergic synapse	6.91 x 10^−5^	26	10	ND
Glutamatergic synapse	9.25 x 10^−5^	22	8	ND
**Wnt signaling pathway**	9.47 x 10^−5^	29	9	12

Notes

ND: not determined.

Pathways in bold are known to be altered with age.

a The number of genes in the pathway that are predicted to be affected by at least one miRNA in the cluster.

b The number of miRNAs predicted to affect at least one gene in the pathway.

c The number of predicted genes shown to decrease with age by RNA‐seq.

To determine whether the predicted targets that are reduced with age are true targets of the mir‐465 family, we transfected the mouse liver cell line AML12 (Wu, Merlino, & Fausto, 1994) with either a plasmid expressing all three mir‐465 family members (pmir465abc) or with a mir‐465c mimic. Expression of the mir‐465 family was increased nearly 100‐fold upon transfection with pmir465abc into AML12 cells (Supporting Information Figure S3). Transfection of the pmir465abc plasmid or the mir‐465c mimic led to a 40% reduction of the GHR mRNA expression, and an approximate 25% reduction of the Kitl and PPP2R3C mRNA expression (Figure 4b). We did not see a significant reduction in expression of Vegfa, Gbn1, Kras, or PTEN mRNA, indicating that these may not be true targets of the mir‐465 family, or that the mir‐465 family may act by inhibiting translation of these target mRNAs instead of initiating degradation.

We focused our investigations on the GHR, since the liver is an important site of GH signaling and the GH signaling pathway is known to be involved in the regulation of normal aging. To determine whether expression of the mir‐465 family leads to a reduction in GHR protein levels, we performed western blotting on AML12 cell extracts and found a 50% decrease in GHR protein levels in cells transfected with the mir‐465 family (Figure 4c). These experiments further substantiate that the mir‐465 family targets the GHR mRNA leading to a reduction in expression of the GHR protein.

Further examination of the mir‐465 target site in the GHR 3’UTR showed that this site is highly conserved across a wide range of mammalian species from shrews to humans (Supporting Information Figure S4), indicating that it may be important for regulating GHR expression and consequently GH signaling. To verify that the predicted target site of the mir‐465 family is functional, we cloned the 3′UTR of the growth hormone receptor gene into the pmirGlo luminescent reporter plasmid. When the reporter plasmid was cotransfected with a mixture of miRNA mimics containing all three mir‐465 family members into HEK‐293T cells, it led to a 50% reduction in luminescence signal (Figure 4d). Mutation of the target site in the GHR 3′UTR restored luminescence indicating that the predicted site in the GHR 3′UTR is an active site recognized by mir‐465 family members. To determine whether differences exist between the activity of the individual mir‐465 family members, we also transfected mir‐465a, mir‐465b, and mir‐465c mimics independently with the GHR reporter plasmid. We found that each mir‐465 mimic caused a ~50% reduction in luminescence, which was comparable to the reduction seen when all three miRNA mimics were transfected together (Figure 4d). These results indicate that all three mir‐465 family members can target the predicted site in the GHR 3′UTR leading to a reduction in GHR mRNA expression.

### 2.3 The effects of miR‐465 family expression on GH signaling

**Figure 5:**
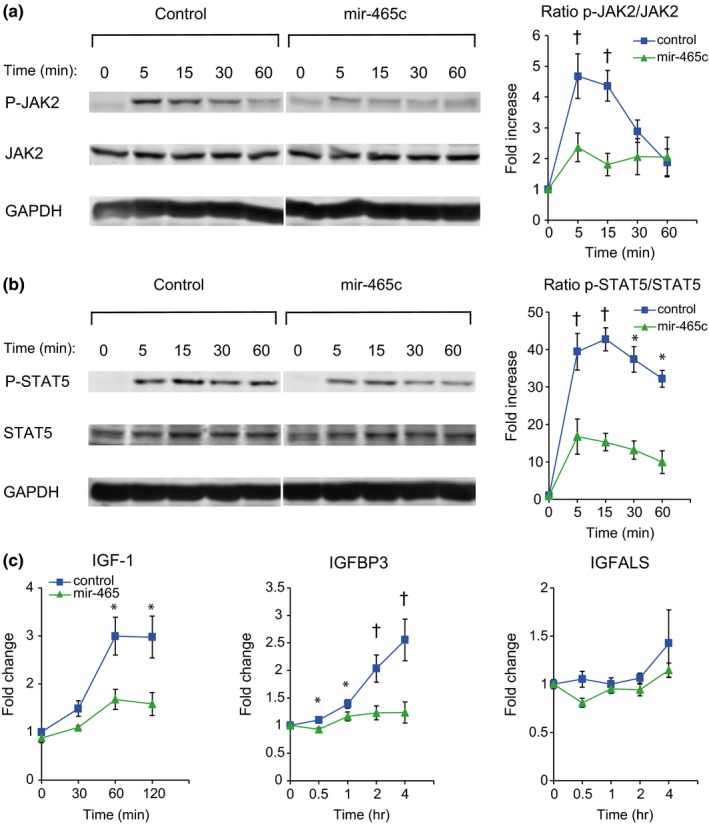
Time course of GH signaling in AML12 cells. The effect of reduced GHR expression by mir‐465c on GH signaling in the cultured mouse liver cell line AML12. (a) Transfection of AML12 cells with a mir‐465c mimic led to reduced JAK2 phosphorylation following GH stimulation. In control cells, the ratio of p‐JAK2/Jak2 increased almost fivefold within 5 min of GH addition and stayed elevated for 15 min following GH treatment. JAK2 phosphorylation was attenuated in cells transfected with a mir‐465c mimic. The ratio of p‐JAK2/JAK2 increased only twofold 5 min. following GH stimulation and remained low over the next 60 min. (b) Phosphorylated active JAK2 phosphorylates STAT5. In control cells, the ratio of p‐STAT5/STAT5 increased 40‐fold within 5 min. following GH stimulation and remained elevated over the next 60 min. AML12 cells transfected with the mir‐465c mimic showed an attenuated response with the ratio of pSTAT5/STAT5 increasing only 15‐fold within 5 min. following GH stimulation and remaining low over the next 60 min. (c) GH signaling results in increased IGF‐1 expression. IGF‐1 expression increased threefold within 60 min of GH stimulation in control cells. Cells transfected with the mir‐465c mimic showed an attenuated response, with IGF‐1 expression increasing 1.5‐fold 60 min. following GH stimulation. Control cells showed a 2‐fold increase in IGFBP3 expression within two hours of GH stimulation. No increase in IGFBP3 expression was observed in cells transfected with the mir‐465c mimic. IGFALS expression did not increase following GH stimulation in control cells or in cells transfected with the mir‐465c mimic. Time = time following GH addition. All error bars represent the standard errors. All *p*‐values were calculated using two‐tailed Student's *t* test. *p < 0.05, ^†^ *p* < 0.01

A reduction in GHR is expected to result in decreased GH signaling. Activation of the GHR leads to phosphorylation of the tyrosine kinase Janus kinase 2 (JAK2), which subsequently phosphorylates the transcription factor signal transducer and activator of transcription 5 (STAT5). Phosphorylated STAT5 then translocates to the nucleus where it activates the transcription of growth hormone response genes including insulin‐like growth factor 1 (IGF‐1), insulin‐like growth factor‐binding protein 3 (IGFBP3), and insulin‐like growth factor acid‐labile subunit (IGFALS). To investigate the effect of mir‐465 family expression on GH signaling, AML12 cells were grown in serum‐free medium for 4 days to allow for expression of liver‐specific genes (Supporting Information Figure S5), followed by transfection with the mir‐465c mimic. GH was added 48 hr posttransfection and activation of the GH signaling pathway was followed for 4 hr. Both control cultures and cultures expressing mir‐465c mimic showed an immediate response to GH by increasing phosphorylation of JAK2 within 5 min (Figure 5a). However, cells containing mir‐465c mimic had a muted response indicated by a twofold increase in JAK2 phosphorylation, as compared to a fivefold increase seen in control cells. A similar reduction in signaling was seen in STAT5 phosphorylation, where control cells showed a much larger increase in STAT5 phosphorylation (43‐fold after 15 min) when compared to cells containing the mir‐465c mimic (15‐fold after 15 min) (Figure 5b). These results show that a reduction in the GHR resulting from the presence of mir‐465c leads to an attenuation of GH signaling in response to GH stimulation.

The primary response to GH signaling is an increase in IGF‐1 expression. To determine whether the reduction in the GHR and the GH signaling response in the presence of mir‐465 leads to reduced IGF‐1 expression, we analyzed IGF‐1 mRNA expression following GH stimulation. We found an attenuation in IGF‐1 mRNA expression in AML12 cells transfected with either pmir465abc or the mir‐465c mimic (Figure 5c). In control cells, IGF‐1 expression increases threefold within 1 hr of GH stimulation. This induction was only 1.5‐fold in cells transfected with pmir465abc or the mir‐465c mimic. IGF‐1 circulates in the plasma as a complex with IGF‐1‐binding protein 3 (IGFBP3) and IGF‐1 acid‐labile subunit (IGFALS). Expression of both of these IGF‐1‐associated proteins increases upon GH stimulation. We saw a twofold increase in IGFBP3 expression in control cells within 2 hr of GH stimulation, which continued to increase at 4 hr post‐GH stimulation. However, in cells transfected with the mir‐465c mimic, there was no increase in expression during this time frame. Contrarily, we did not see a significant increase in IGFALS expression in either control or mir‐465c‐treated cells. These results indicate that the reduction in GHR in the presence of mir‐465 family members leads to a reduction in IGF‐1 and IGFBP3 expression in response to GH stimulation.

## 3 DISCUSSION

We performed small transcriptome analysis to identify changes in small RNA expression in aged male mouse liver. We found 56 miRNAs that changed expression levels with age. The most prominent change we found was the upregulation of a cluster of 18 miRNA genes present in a 60‐kb region of the X‐chromosome. This derepression was most pronounced in aged mouse liver, but also occurred in other tissues including skeletal muscle and brain. This cluster of 18 miRNA genes is expressed in the testes and the majority of the miRNAs show minimal expression in other tissues (Landgraf et al., 2007; Ro et al., 2007). The derepressed miRNAs include members of the mir‐465 family: one copy of mir‐465a and two copies each of mir‐465b and c. These five miRNAs appear to be the result of duplication events as they only vary by 1–3 nucleotides, have the same seed sequence, and are predicted to recognize the same mRNA targets. Here, we show that all members of the mir‐465 family target the 3′UTR of the GHR mRNA equally, indicating that all three mir‐465 family members have equal specificity for the GHR mRNA 3′UTR. We believe that this is the first report on the functionality of this family of miRNAs. While the data presented here are specific for male mice, we expect to see similar, if not more prominent, upregulation of the X‐chromosomal miRNA cluster in female mice due to loss of repression of sequences on the inactivated X‐chromosome with age. Telomere shortening, which is associated with organismal aging, was shown to contribute to a loss of H3K27me3 silencing and an upregulation of sequences on the inactivated X‐chromosome in female mice (Schoeftner et al., 2009). Future in vivo studies will investigate gender‐specific differences in the upregulation of these miRNAs.

The somatotrophic axis in mammals consists of growth hormone‐releasing hormone (GHRH), GH, and IGF‐1 that work in concert to regulate cell survival and growth. The principal site of GH action is the liver, where it binds to the GHR initiating a signaling cascade through the JAK/STAT pathway resulting in IGF‐1 production. In mammals, levels of circulating GH and IGF‐1 begin to decline in early adulthood and continue to decline throughout the lifespan in a process called somatopause (Bartke, 2008). A reduction in circulating IGF‐1 in adulthood is a major risk factor for the development of a number of age‐associated pathologies and diseases, including diabetes, osteoporosis, sarcopenia, cardiovascular disease, dementia, and Alzheimer's disease (Bartke et al., 2016; Barzilai et al., 2012; Laughlin, Barrett‐Connor, Criqui, & Kritz‐Silverstein, 2004; Lombardi et al., 2005; Toth et al., 2015; Westwood et al., 2014). GH signaling also plays a significant role in regulating hepatic lipid metabolism. A reduction in GH signaling, through either GH deficiency or reduced GHR activity, leads to an upregulation of the fatty acid transporter Cd36, and several genes involved in lipogenesis (*Fasn, Scd1*) resulting in hepatic accumulation of triglycerides and lipid, leading to the development of nonalcoholic fatty liver disease (NAFLD; reviewed in Kaltenecker et al., 2018). The data presented here suggest that the increase in expression of the mir‐465 family could contribute to the reduction in circulating IGF‐1 and to the onset of NAFLD with age.

The mechanism behind the derepression of the cluster of miRNAs is currently unknown and is a direction of future study. Recent work from our laboratory and others established that a global change in chromatin structure occurs with age, characterized by heterochromatic regions of the genome becoming relatively more open and the derepression of repetitive elements present in these regions (Criscione et al., 2016; Criscione, Zhang, Thompson, Sedivy, & Neretti, 2014; De Cecco, Criscione, Peckham, et al., 2013; De Cecco, Criscione, Peterson, et al., 2013; Kreiling et al., 2011; Swanson, Manning, Zhang, & Lawrence, 2013; Wood et al., 2010). In addition, we found that lamin‐associated domains dissociate from the nuclear lamina in senescent human diploid fibroblasts (HDF) indicating a loss of repression (De Cecco, Criscione, Peckham, et al., 2013). We found that the cluster of miRNAs is located in a 60‐kb gene‐poor region of the X‐chromosome that contains high numbers of repetitive elements and is enriched in beta‐lamin interactions, indicating that it is part of a heterochromatic lamin‐associated domain. Therefore, the age‐associated relaxation of heterochromatin may lead to expression of these miRNAs, which could subsequently affect the major signaling pathways that are known to be altered with age.

We began to see an increase in expression of mir‐465 family members at 12 months of age, and expression continued to increase until 24 months of age. We found that an increase in mir‐465 family expression in a mouse liver cell line by transfection of mir‐465 mimics led to a reduction in GHR at both the mRNA and protein levels, suggesting that these miRNAs act by inducing mRNA degradation rather than inhibiting translation. The reduction in GHR resulted in reduced IGF‐1 and IGFBP3 expression. Contrarily, we did not see an increase in IGFALS, as would be expected to result from GH stimulation (Dai, Scott, & Baxter, 1994; Ooi, Cohen, Tseng, Rechler, & Boisclair, 1997). However, it has been reported that induction of IGFALS following GH stimulation in cultured cell lines is not detected and it is hypothesized that altered chromatin structure in cultured cell lines may contribute to this phenotype (Dai et al., 1994; Ooi et al., 1997). The reduction of IGF‐1 and IGFPB3 expression indicates that dysregulation of mir‐465 family expression with age may contribute, at least in part, to the reduction in circulating IGF‐1 with age in vivo through its action on GHR expression. Future studies in vivo will help elucidate the effects of mir‐465 family expression on the somatotrophic axis and healthspan.

The role of GH/IGF‐1 signaling on healthspan and lifespan has been studied in a wide range of organisms from invertebrates to humans (Bartke et al., 2016; Barzilai et al., 2012; Brown‐Borg, 2015). Interestingly, there is a high degree of conservation in the GH/IGF‐1 signaling pathway across species in both its function and effects on healthspan and lifespan. The mir‐465 family target site in the 3′ UTR of the GHR mRNA is highly conserved across mammals, indicating that it is likely important for regulation of GHR expression (Supporting Information Figure S4). While the mir‐465 family has only been identified in rodents to date, the conservation of the target site in the GHR mRNA 3′UTR across mammals, including humans, indicates that there are likely unidentified miRNAs or other regulatory elements that are involved in regulation of GHR expression at this site.

We found two additional targets that are downregulated in the presence of mir‐465 family. Kitl, also known as stem cell factor, is a growth factor that binds and activates the receptor tyrosine kinase c‐Kit, initiating PI3K signaling. c‐Kit signaling has been shown to promote proliferation, migration, survival, and differentiation in a wide range of cell types including hematopoietic progenitors, melanocytes, germ cells, interstitial cells of Cajal, neural progenitors, vascular smooth muscle cells, and epithelial progenitor cells (reviewed in Lennartsson & Ronnstrand, 2012). In addition, mutations in Kitl or c‐Kit have been documented in a large number of cancers (Sanger Institute Catalogue of Somatic Mutations in Cancer, 2018). However, c‐Kit signaling has not been investigated in liver in the context of aging. Given its role in cell survival and differentiation, a reduction of Kitl expression with age could lead to dysregulation of hepatocyte function.

We also found PPP2R3C is downregulated by the mir‐465 family. PPP2R3C is a regulatory subunit of protein phosphatase 2 (PP2A) and serine/threonine‐protein phosphatase 5 (PP5; Kono et al., 2002). These phosphoprotein phosphatases are highly conserved in eukaryotes (Brautigan, 2013). PP2A and PP5 regulate signal transduction pathways that control most cellular processes including DNA replication, transcription, translation, metabolism, cell cycle progression, cell division, development, and apoptosis (Janssens & Goris, 2001; Kono et al., 2002; Perrotti & Neviani, 2013). In addition, PPP2R3C regulates P‐glycoprotein (P‐gp), an efflux pump that removes toxic compounds from the cell, including chemotherapy drugs making them less effective (Katayama, Yamaguchi, Noguchi, & Sugimoto, 2014). The role PPP2R3C plays in liver homeostasis and changes that occur with age have not been investigated, but given the wide range of cellular processes it regulates it is expected that a reduction in expression could alter cellular function.

Only one other miRNA in the cluster, mir‐470, has been shown to be expressed outside of the testes. It is expressed during differentiation of embryonic stem cells where it targets Nanog and Oct4 expression (Tay, Zhang, Thomson, Lim, & Rigoutsos, 2008). It is also expressed in the central nervous system. In the adult brain, mir‐470 expression is tied to regulation of neuronal firing (van Spronsen et al., 2013). Taken together with our finding that mir‐470 and several other members of the X‐chromosome cluster are upregulated in the brain with age (Supporting Information Figure S2), and that the miRNAs in the cluster are predicted to influence axon guidance, GABAergic synapse, dopaminergic synapse, and glutamatergic synapse pathways (Table 1), the upregulation of these miRNAs may be important in age‐associated changes in neuronal function. In addition, mir‐470 is expressed in the hippocampus of the Ames dwarf long‐lived mouse where its expression leads to a >30% reduction in IGF‐1 receptor (Liang et al., 2011). These results are interesting, when combined with the results presented here they suggest that the cluster of miRNAs may work together to regulate the somatotrophic axis. This hypothesis is further strengthened when you consider that bioinformatic analyses predict 14 of the miRNAs have potential targets in 69 genes involved in PI3K‐Akt signaling (Table 1). These include the PI3K 3’UTR that is predicted to be recognized by mir‐743a and mir‐871, the AKT3 3’UTR that is predicted to be targeted by mir‐883b, and the IGF‐1 3’UTR that is predicted to be targeted by mir‐743a, mir‐871, and mir‐881. Since all 18 of the miRNAs are upregulated as a cluster, it is possible that they work together in concert to regulate various aspects of PI3K‐Akt signaling. Therefore, dysregulation of the X‐chromosomal cluster of miRNAs with age may have broader reaching consequences by attenuating several segments of this signaling pathway. It is known that PI3K‐Akt signaling is altered in many of the diseases associated with aging. Future studies involving additional miRNAs found in the X‐chromosome cluster will provide insight into the role that these miRNAs play in both normal aging and the onset of age‐associated pathologies.

## 4 EXPERIMENTAL PROCEDURES

### 4.1 Mouse tissue

Mouse tissues of strain C57BL/6 were collected in‐house from male mice obtained from the NIA Aged Rodent Colonies (https://www.nia.nih.gov/research/dab/aged-rodent-colonies-handbook).

### 4.2 Cell lines

The human embryonic kidney cell line HEK‐293T and the mouse hepatocyte cell line AML12 (both from ATCC, Manassas, VA, USA) were used in this study. For detailed cell culture procedures, see Supporting Information Data S1.

### 4.3 High‐throughput sequencing

The small RNA fraction was isolated from young (5 months), old (24 months), and very old (36 months) mouse liver and sequenced on the Illumina HiSeq2000. We previously published the RNA‐seq datasets (De Cecco, Criscione, Peterson, et al., 2013). See Supporting Information Data S1 for detailed protocols for both datasets.

### 4.4 Luminescence assay

The full‐length 3′UTR of the GHR gene was cloned into the pmirGLO reporter plasmid (Promega, Madison, WI, USA) at the 3′ end of the firefly luciferase gene (pGloGHR). Site‐directed mutagenesis was used to eliminate the potential mir‐465 target site in pGloGHR. HEK‐293T cells were cotransfected with miRNA mimics corresponding to mmu‐mir‐465a, mmu‐mir‐465b, mmu‐mir‐465c, or cel‐mir‐39 (Exiqon, Woburn, MA, USA) and either pGloGHR or the empty vector. After 48 hr, Firefly and Renilla luciferase activity was detected using the DualGlo Luciferase Assay System (Promega) according to the manufacturer's protocol. Detailed protocols can be found in the Supporting Information Data S1.

### 4.5 miRNA expression plasmid and mimics

The coding genes of the three members of the mir‐465 family were cloned into the mammalian expression vector pcDNA3.1 under the control of the CMV promoter to create pmir465abc. Full descriptions of the primers used (Supporting Information Table S1) and the cloning procedure can be found in the Supporting Information Data S1.

### 4.6 GH stimulation

AML12 cells were cultured for 96 hr in culture medium without serum to stimulate the expression of liver‐specific genes including the GHR (Supporting Information Figure S5). The medium was replaced with serum‐free culture medium containing 500 ng/ml mouse growth hormone (GenScript, Piscataway, NJ, USA). See the Supporting Information Data S1 for more details.

### 4.7 Quantitative real‐time PCR

Quantification of miRNA expression was performed using cDNA made following the procedure outlined in Balcells, Cirera, and Busk (2011). Details on the RNA isolation, cDNA synthesis, primer design, and cycling parameters can be found in the Supporting Information Data S1.

### 4.8 Quantitative immunoblotting

Following GH treatment, cells were harvested, separated by SDS‐PAGE, and transferred onto Immobilon‐P membranes (MilliporeSigma, Burlington, MA, USA). Membranes were probed with the appropriate primary and secondary antibodies (Supporting Information Table S2). Signals were detected using the Li‐Cor Odyssey Clx Infrared Imaging System (Li‐Cor Biosciences, Lincoln, NE, USA) and analyzed either with the gel analysis component of ImageJ open source software (https://imagej.nih.gov/ij/) or with the Li‐Cor Odyssey analysis software. A detailed protocol can be found in the Supporting Information Data S1.

### 4.9 Antibodies

Primary and secondary antibodies and dilutions used in this study are outlined in Supporting Information Table S2. Secondary antibodies for immunoblotting were labeled with Odyssey IRDye 680CW or 800CW (Li‐Cor Biosciences).

## CONFLICT OF INTEREST

The authors declare no conflicts of interest.

## AUTHORS’ CONTRIBUTIONS

J.A.K. conceived and designed the study, performed experiments, analyzed data, and wrote the manuscript. A.E.E. performed experiments and analyzed data. B.K., I.M.C.S., G.G.‐M., and A.C.Z. performed experiments.
