# Supplementary material for: The mir‐465 family is upregulated with age and attenuates growth hormone signaling in mouse liver
Source: Aging Cell. 2019 Jan 13;18(2):e12892. doi: 10.1111/acel.12892 (PMC6413667; doi:10.1111/acel.12892)
Supplement: Supplementary file 6 [file ACEL-18-e12892-s006.docx]

**Supplemental Information**

**Supplemental Experimental Procedures**

**Cell lines**

The human embryonic kidney cell line HEK-293T was cultured in Dulbecco’s Modified Eagle’s Medium (DMEM) supplemented with 10% fetal bovine serum (Hyclone), 20 mM L-glutamine and 1% penicillin/streptomycin (Sigma Aldrich). The mouse hepatocyte cell line AML12 was cultured in a 1:1 mixture of DMEM and Ham's F12 medium supplemented with 10% fetal bovine serum, 0.005 mg/ml insulin, 0.005 mg/ml transferrin, 5 ng/ml selenium, 40 ng/ml dexamethasone, 20 mM L-glutamine and 1% penicillin/streptomycin. Cultures were maintained using a 1:6 subculture regimen and incubated at 37°C in an atmosphere of 95% air and 5%CO_2_. Cells were split into fresh medium after reaching 80% confluence. For the induction of liver specific genes AML12 cells were split 1:32 in complete medium (above) and allowed to attach to the plate overnight. The medium was replaced with the complete medium without fetal bovine serum. Cells were cultured for 4 additional days to induce liver specific gene expression, Figure. S5. Transfections were carried out on day 4 post-splitting medium change. After 48 hours post-transfection cells were approximately 50-60% confluent, which allowed for GH stimulation. Cells at a higher confluence had a poor GH response.

**High throughput sequencing**

The small RNA fraction (<200 nt) was isolated from 500-800 mg of liver from young ( 5 month), old (24 month) and very old (36 month) mouse liver using the MirVana kit (ThermoFisher, Waltham, MA, USA) according to the manufacturer’s specifications. Four biological replicates from each condition were included in the experiment. Small RNA samples were quantified using a NanoDrop spectrophotometer and the size range and purity of each sample was confirmed on a Bioanalyzer using the Eukaryote Total RNA Nano chip. Sequence libraries were created with NEXTflex Small RNA Sequencing Kit (Bioo Scientific, Austin, TX, USA) following the protocol provided by the company. Briefly, 1.2-2.6 ug of purified small RNA was used for each sample. Universal adapters were added to the 3’ end using AIR Ligase (Bioo Scientific, Austin, TX, USA) and to the 5’ end using T4 ligase, both provided with the kit. The ligated RNA was cleaned up with the RNA Clean & Concentrator-5 kit (Zymo Research, Irvine, CA, USA) following the instructions provided. Strand-specific cDNA was transcribed using a primer specific for the 3’ adapter and M-MuLV reverse transcriptase provided in the kit. The resulting single stranded cDNA was amplified and barcoded by PCR for 15 cycles using primers specific for the 5’ and 3’ adapters. The reactions were subsequently purified by electrophoresis on a 15% polyacrylamide gel, retaining the 150-350 base pair fragments. The purified libraries were sequenced on the Illumina HiSeq2000 by the Brown University Genomics Core to generate single-end 50 nucleotide reads. Sequencing reads were groomed using the Galaxy platform (Blankenberg et al., 2010; Giardine et al., 2005; Goecks et al., 2010). Putative mature and precursor miRNAs were identified and quantified using MirDeep (Friedlander et al., 2008). Differential expression and statistical significance were determined using EdgeR (McCarthy et al., 2012; Robinson et al., 2010). Changes in expression were deemed significant if the p-value and false discovery rate (FDR) were ≤ 0.05. The biological targets of the miRNAs were predicted using DIANA Tools micro-CDS and mirPath software (<http://diana.imis.athena-innovation.gr/DianaTools>, (Vlachos et al., 2012)), and Target Scan (<http://www.targetscan.org/vert_61/>) (Garcia et al., 2011; Grimson et al., 2007; Lewis et al., 2005). The genomic location of the miRNAs was visualized with **UCSC Genome Browser (Kent et al., 2002)** using mouse genome assembly mm9 ([http://genome.ucsc.edu/](http://genome.ucsc.edu/index.html)).

RNA-seq was performed as previously described (De Cecco et al., 2013). Briefly, 30-50 mg of liver was used for RNA extraction. Poly-adenylated mRNA was prepared using two consecutive purifications with oligo-dT magnetic beads, following the mRNA Direct Dynabeads Kit protocol provided by the manufacturer (ThermoFisher, Waltham, MA, USA). The eluted mRNA was quantified using the Qubit 2.0 RNA HS Assay Kit (ThermoFisher, Waltham, MA, USA). 200 ng of mRNA was fragmented using the RNA Fragmentation Kit (ThermoFisher, Waltham, MA, USA). Fragmented mRNA was precipitated with isopropanol and glycogen (ThermoFisher, Waltham, MA, USA), resuspended in RNAse-free water, and reverse transcribed using the SuperScript III First Strand Kit (ThermoFisher, Waltham, MA, USA). A non-strand-specific second strand DNA synthesis was performed using the Second Strand Buffer (New England Biolabs, Ipswich, MA, USA)), DNA polymerase I and RNAse H. 20 ng of double-stranded cDNA per sample was end repaired with the End-It DNA End Repair Kit (Epicentre, Madison, WI, USA) according to the provided protocol. DNA was purified using Agencourt AMPure XP Paramagnetic Beads (Beckman Coulter, Atlanta, GA, USA), and eluted in molecular grade water. dATP was added to the DNA ends (Kozarewa & Turner, 2011), and after another DNA purification, pre-annealed adapters were ligated to each sample (Quail et al., 2008). Ten cycles of PCR amplification were performed using Phusion High-Fidelity DNA Polymerase (New England Biolabs, Ipswich, MA, USA)), and the libraries were agarose gel purified retaining fragments in the range of 200-500 base pairs. RNAseq libraries were sequenced on an Illumina HighSeq 2000 instrument by the Brown University Genomics Core to generate single-end 50 nucleotide reads. The sequencing data were uploaded to the Galaxy platform (main.g2.bx.psu.edu/) and mapped with TopHat against the mouse genome (mm10) (Trapnell et al., 2009). Cufflinks was used to estimate gene transcript abundance in reads per kilobase per million mapped reads (RPKM) and edgeR was used for analysis for differential gene transcript expression (McCarthy et al., 2012; Robinson et al., 2010).

**Luminescence assay**

The full length 3’UTR of the GHR gene was amplified from genomic mouse DNA with the Phusion High-Fidelity DNA Polymerase (New England Biolabs, Ipswich, MA, USA) using primers that introduced a NheI site at the 5’ end and an XbaI site at the 3’ end (Table S1). The PCR cycling parameters were: 98°C for 2 min.; 5 cycles of 98°C for 10 sec., 55°C for 30 sec., 72°C for 2.5 min.; 30 cycles of 98°C for 10 sec., 60°C for 4 min., 72°C for 2.5 min (Elzein & Goodyer, 2014). The PCR product was cloned into the pmiRGLO reporter plasmid (Promega, Madison, WI, USA) at the 3’ end of the firefly luciferase gene (pGloGHR). Site-directed mutagenesis was used to eliminate the potential mir-465 target site in pGloGHR using the Q5 Site-Directed Mutagenesis Kit (New England Biolabs, Ipswich, MA, USA) using the GHRmut primer pair (Table S1**)** following the manufacturer’s instructions (pGloGHRmut).

HEK-293T cells were plated in 96 well dishes at a density of 7000 cells per well and cultured for 24 hours at 37°C. Cells were co-transfected with 15 pmoles of miRNA mimics corresponding to mmu-mir-465a, mmu-mir-465b, mmu-mir-465c, or cel-mir-39 (Exiqon, Woburn, MA, USA) and either a reporter plasmid containing the GHR 3’UTR or the empty vector. Transfections were done using Fugene HD (Promega, Madison, WI, USA) at a 3:1 ratio of the transfection reagent to nucleic acid following the manufacturer’s instructions. Transfected cells were incubated at 37°C for 48 hours. Firefly and Renilla luciferase activity was detected using the DualGlo Luciferase Assay System (Promega, Madison, WI, USA) according to the manufacturer’s protocol. Luminescence signals were read on a Modulus Luminometer (Promega, Madison, WI, USA). The Renilla luciferase value was used to normalize the firefly luciferase signal. Significant differences were determined using a 2-tailed Student’s t-test.

**miRNA expression plasmid and mimics**

The coding genes of the three members of the mir-465 family were cloned into the mammalian expression vector pcDNA3.1 under the control of the CMV promoter to create pmir465abc. For each mir-465 gene PCR primers containing restriction enzyme recognition sites (Table S1) were designed using Primer BLAST software ([www.ncbi.nlm.nih.gov/tools/primer-blast/](http://www.ncbi.nlm.nih.gov/tools/primer-blast/)) to amplify ~200 bp genomic fragments containing coding gene plus 70 bp upstream and downstream of each miRNA gene. PCR was performed on 10 ng of liver genomic DNA with the following parameters: 2 min at 95°C; 5 cycles of 95°C for 30 sec., 55°C for 30 sec, 72°C for 30 sec.; 35 cycles of 95°C for 30 sec., 60°C for 30 sec., 72°C for 30 sec.; ending with a 10 min 72°C extension. The resulting PCR products were purified using the MinElute PCR Purification kit (Qiagen, Valencia, CA, USA) following the manufacturer’s instructions. The PCR products were digested with the appropriate restriction enzymes (New England Biolabs, Ipswich, MA, USA)), cleaned up with the MinElute Reaction Cleanup kit (Qiagen, Valencia, CA, USA) and cloned in tandem into pcDNA3.1. MicroRNA mimics for mmu-mir-465a-5p, mmu-mir-465b-5p, and mmu-mir-465c-5p were purchased from Exiqon (Woburn, MA, USA). Negative Control 5 miRCURY LNA miRNA Mimic (GAUGCUACGGUCAAUGUCUAAG) was used as a control (Exiqon, Woburn, MA, USA).

**GH stimulation**

AML12 cells were cultured for 96 hours in culture medium (described above) without serum to stimulate the expression of liver specific genes including the GHR **(**Figure S5). Cells were transfected with pmiR465abc using Fugene HD (Promega, Madison, WI, USA) at a 6:1 ratio of transfection reagent to µg of nucleic acid, or the mir-465 mimics at a 4:1 ratio of Fugene HD transfection reagent (Promega, Madison, WI, USA) to µg of nucleic acid. Transfected cells were cultured for 48 hours in serum-free medium, and then the medium was replaced with serum-free culture medium containing 500 ng/ml mouse growth hormone GenScript, Piscataway, NJ, USA). At each time-point cells were harvested by scraping into Trizol reagent for RNA isolation, or into Laemmli buffer (50mM Tris HCL pH 6.8, 2% SDS, 100 mM DTT, 20 mM sodium orthovanadate, 10% glycerol, 0.2% bromophenol blue) for Western blot analysis.

**Quantitative real-time PCR**

RNA was isolated using a combination of Trizol reagent and the RNeasy kit (Qiagen, Valencia, CA, USA). 30-50 mg of tissue was homogenized in 1 ml Trizol and extracted with 200µl of chloroform. Following centrifugation at 12,000 x g for 15 min. the RNA in the aqueous phase precipitated by adding 500 µl isopropanol. The precipitated RNA in solution was loaded onto the RNeasy column and purified according to the manufacturer’s instructions. The RNA was quantified using a NanoDrop spectrophotometer. For quantification of mRNA expression 1 μg of total RNA was transcribed into cDNA in 50 μL reactions using random hexamers and the TaqMan kit (Applied Biosystems, Foster City, CA, USA), according to the manufacturer's protocol. A volume of 0.1-1.5 μL of this reaction was used in subsequent qPCR reactions.

For quantification of miRNA expression cDNA was made following the procedure outlined in Balcells et al. (2011). A quantity of 1 ug of total RNA was polyadenylated at the 3’ end by incubating for 30 min. at 37°C with 0.25 U/µl polyA polymerase (New England Biolabs, Ipswich, MA, USA), 1 mM ATP (New England Biolabs, Ipswich, MA, USA)), 5.5 mM MgCl_2_ in 1x reverse transcription (RT) buffer from the TaqMan kit (Applied Biosystems, Foster City, CA, USA) in a 25 µl volume. A degenerate RT-primer was added to a final concentration of 200 nM and allowed to anneal at 60°C for 5 min. Finally, the RNA was reverse transcribed to cDNA by incubating for 1 hour at 42°C with 500 µM (each) dNTP mix (Applied Biosystems, Foster City, CA, USA), 1.25 U/µl MultiScribe reverse transcriptase (Applied Biosystems, Foster City, CA, USA), 5.5 mM MgCl_2_ in 1x RT buffer in a final volume of 50 µl.

qPCR was performed using the SYBR Green system (Applied Biosystems, Foster City, CA, USA) on an ABI ViiA 7 Real Time System instrument (Applied Biosystems, Foster City, CA, USA), according to the manufacturer's specifications. Primers for mRNAs were designed using the Primer-BLAST software. Primers for miRNA quantification were designed using miRprimer (Busk, 2014). Primer sequences can be found in Table S1**.** The forward and reverse primers were used at a concentration of 300 nM each. GAPDH, HPRT, Rn18S, and Actb were used as normalization controls for mRNA expression. U6 was used as a normalization control for miRNA expression. Significance was determined using 2-tailed Student’s t-test.

**Quantitative immunoblotting**

Following GH treatment cells were harvested by scraping into Laemmli buffer (50mM Tris HCL pH 6.8, 2% SDS, 100 mM DTT, 20 mM sodium orthovanadate, 10% glycerol, 0.2% bromophenol blue) and boiled for 5 min. Protein concentrations were determined using the Qubit Protein Assay Kit on a Qubit 2.0 fluorometer (ThermoFisher, Waltham, MA, USA) according to the manufacturer’s protocol. 45-50 µg of protein was loaded per lane, separated by SDS-PAGE, and transferred onto Immobilon-P membranes (MilliporeSigma, Burlington, MA, USA). Membranes were blocked with 5% milk in Tris-buffered saline (TBS) for 1 hr, and then probed with the appropriate primary antibody (Table S2) overnight at 4°C. Membranes were subsequently washed in TBS and incubated with secondary antibodies labeled with Odyssey IRDye 680CW or 800CW ((Li-Cor Biosciences, Lincoln, NE, USA). Signals were detected using the LiCor Odyssey Clx Infrared Imaging System (Li-Cor Biosciences, Lincoln, NE, USA), and analyzed either with the gel analysis component of ImageJ open source software (<https://imagej.nih.gov/ij/>) or with the LiCor Osyssey analysis software. Signals were normalized to GAPDH signals. Statistical significance was determined using 2-tailed Student’s t-test.

**Supplemental Figure Legends**

**Figure S1: Individual Variation in Expression of the miRNAs in the X-Chromosome Cluster.**

A representative experiment showing qPCR analysis of the expression of 8 members of the miRNA cluster at 6, 12, 18, 24, and 36 months of age. Results for individual animals are shown (blue) along with the average for each age group (red). Note the large variation in miRNA expression between individual animals at 24 month and 36 month of age. Although there is a large variation in expression between individual animals for each miRNA, the relative expression levels of all 8 miRNAs are consistent for each animal.

**Figure S2: Expression of miRNAs in Skeletal Muscle, Brain, and Cultured Mouse**

**Tail Fibroblasts.**

The increase in expression of the X-chromosomal miRNA cluster was not limited to liver. A) Expression of these miRNAs increases 4-9 fold at 24 months of age in skeletal muscle. Expression was reduced at 36 months of age, but was still around 2 fold higher than at 5 months of age. n=3. B) In the brain, expression of some of the miRNAs increased around 1.5 fold by 36 months of age. Most of the increases were seen in miRNAs located at the 5’ end of the cluster, whereas those found at the 3’ end were not changed. n=3. C) There is a modest increase in expression (1.7-3 fold) of the miRNAs in senescent mouse-tail fibroblasts. n=6. Error bars represent the standard errors. All p-values were calculated using the 2-tailed Student's t-test. * = p<0.05, † = p<0.01.

**Figure S3: Transfection Efficiency of the AML12 cell line.**

AML12 cells were transfected with pmir465abc and expression of mir-465b was determined by qPCR after 48 hours. EV – empty vector (pcDNA3.1). The transfection mixtures were as follows: 1.5 : 1 = 1.5 µl of Fugene HD to 1 µg of DNA. 3.0 : 1 = 3.0 µl of Fugene HD to 1 µg of DNA. 4.0 : 1 = 4.0 µl of Fugene HD to 1 µg DNA. 6.0 : 1 = 6.0 µl of Fugene HD to 1 µg DNA. The 6.0 : 1 ratio had the highest increase in mir-465b expression levels (around 90 fold) and was used for the transfection experiments. n=2. The error bars represent the standard deviations. All p-values were calculated using the 2-tailed Student's t-test. * = p<0.05, † = p<0.01.

**Figure S4: The mir-465 Family Target Site in the GHR 3’UTR is Conserved in Mammals.**

The predicted mir-465 target site is conserved across a wide range of mammals (TargetScan, <http://www.targetscan.org/mmu_61>) (Friedman et al., 2009). The white area indicates the region of conservation. Conservation is not seen outside of the mammalian linage.

**Figure S5: Serum-starvation induces liver specific gene expression in AML12 cells.**

AML12 cells were plated on tissue culture dishes and allowed to attach for 24 hours. The DMEM/F12 media + 10% fetal calf serum was replaced with complete medium lacking serum. The expression of the GHR and albumen were followed for one week to determine the time-frame for liver specific gene induction. We found a >2-fold increase in expression of the GHR at 4 days post-media change. The expression of albumen increased much more significantly with over 100-fold increase after 4 days that continued to rise at 7 days. We used a 4 day serum-starvation protocol in the GH stimulation experiments since this time showed the maximum induction of GHR. n=2. The error bars represent the standard deviations. All p-values were calculated using the 2-tailed Student's t-test. † = p<0.01.

| **Table S1: Primers used in this study.** | |
| --- | --- |
| **Primer Name** | **Sequence** |
| ***miRNA quantification:*** | |
| Universal RT | CAGGTCCAGTTTTTTTTTTTTTTTVN |
| MIR465BSF | CGCAGTATTTAGAATGGTGCTGA |
| MIR465BCSR | GGTCCAGTTTTTTTTTTTTTTTCAGA |
| MIR465CSF | GCAGTATTTAGAATGGCGCTGA |
| MIR883AF | CGCAGTGCTGAGAGAAGTAGCAG |
| MIR883AR | AGGTCCAGTTTTTTTTTTTTTTTGTAA |
| MIR470F | AGTTCTTGGACTGGCACTGGT |
| MIR470R | CAGGTCCAGTTTTTTTTTTTTTTTACTC |
| MIR743AF | GCAGTATTCAGATTGGTGCCTG |
| MIR743AR | AGGTCCAGTTTTTTTTTTTTTTTATGA |
| MIR878F | CCGCAGTATCTAGTTGGATGTCA |
| MIR878R | GGTCCAGTTTTTTTTTTTTTTTGTCT |
| MIR471F | CGCGCAGTACGTAGTATAGTGCTTT |
| MIR471R | GGTCCAGTTTTTTTTTTTTTTTGTGA |
| U6F | CGATACAGAGAAGATTTAGCATGGC |
| U6R | TCCAGTTTTTTTTTTTTTAAAAATATGG |
| ***miRNA target quantification:*** | |
| GHRF | GTGCAACCTGATCCACCCAT |
| GHRR | CTCCACGAATCCCGGTCAAA |
| PPP2R3CF | CTTAAGGAGTACAGCGCCCTTC |
| PPP2R3CR | ACAACACTGGCGTAGCTTCTG |
| KitlF | TGCTGGTGCAATATGCTGGA |
| KitlR | CCCGCAGATCTCCTTGGTTT |
| VegfaF | GCAGCGACAAGGCAGACTAT |
| VegfaR | AACCTCCTCAAACCGTTGGC |
| Gnb1F | GACTTACATGTGTGGGAAACCAG |
| Gnb1R | ACGAGCATCTCTAATTTGGTTCTTC |
| KrasF | GCAATGAGGGACCAGTACATGA |
| KrasR | CCTACCAGGACCATAGGCACA |
| PTENF | AGCTTCTGCCATCTCTCTCCT |
| PTENR | TCTGCAGGAAATCCCATAGCAATA |
| GAPDHF | AGGTTGTCTCCTGCGACTTC |
| GAPDHR | TGTCATACCAGGAAATGAGCTTG |
| HPRTF | TCCCAGCGTCGTGATTAGCGATG |
| HPRTR | GGCCACAATGTGATGGCCTCCC |
| Rn18SF | TGCGGGCCATAAGCTTGCGT |
| Rn18SR | AGGGCCGTGGGCCTCACTAA |
| ActbF | GTCGAGTCGCGTCCACC |
| ActbR | GTCATCCATGGCGAACTGGT |
| ***Cloning:*** | |
| GhrLNheIF | ATTAATGCTAGCCCTTTCCTATCTTTAATGGCAAGGGA |
| GhrLXbaIR | TCCGATTCTAGAATTTATTGAACTTTGAAGGGAAAAATCCAC |
| MIR465cKpnIF | CGATTAGGTACCCACTTTACATGTACTTATAGATACTC |
| MIR465cHinDIIIR | CGATTAAAGCTTTGATCAAGCTAAGAAGACTG |
| MIR465bHinDIIIF | CGATTAAAGCTTGCACATTACATGTACCTACAGA |
| MIR465bBamHIR | CGATTAGGATCCGATGAAGCTAACAAGACTGT |
| MIR465aBamHIF | CGATTAGGATCCGAAGGCACTTTTCATGTACC |
| MIR465aAflIIR | CGATTACTTAAGTGAGATAAAACTAACAAGACTGT |
| GHRmutF | GGGCCTCAATCAGTTCCAGAATG |
| GHRmutR | CCCCTATTTTTGGCTATAGAAACG |

| **Table S2: Antibodies used in this study.** | | | |
| --- | --- | --- | --- |
| **Antibody** | **Dilution** | **Company** | **Catalog number** |
| *Primary antibodies:* | | | |
| GHR Antibody | 1:200 | Biorbyt | orb017R |
| GAPDH Antibody | 1:1000 | Sigma Aldrich | G8795 |
| Phospho-Jak2 (Tyr1007/1008) Antibody | 1:1000 | Cell Signaling Technology | 3771S |
| Jak2 (D2E12) Antibody | 1:1000 | Cell Signaling Technology | 3230S |
| Phospho-Stat5 (Tyr694) Antibody | 1:200 | Cell Signaling Technology | 9351S |
| Stat5 Antibody | 1:200 | Cell Signaling Technology | 9363S |
| *Secondary antibodies:* | | | |
| Goat α-Mouse IgG 800CW | 1:1000 | LI-COR Biosciences | 926-32210 |
| Goat α-Rabbit IgG 680CW | 1:1000 | LI-COR Biosciences | 926-32211 |

**Supplemental Literature Cited**

Blankenberg, D., Von Kuster, G., Coraor, N., Ananda, G., Lazarus, R., Mangan, M., . . . Taylor, J. (2010). Galaxy: A web-based genome analysis tool for experimentalists. *Curr Protoc Mol Biol, Chapter 19*, Unit 19 10 11-21. doi:10.1002/0471142727.mb1910s89

Busk, P. K. (2014). A tool for design of primers for microrna-specific quantitative rt-qpcr. *BMC Bioinformatics, 15*, 29. doi:10.1186/1471-2105-15-29

De Cecco, M., Criscione, S. W., Peterson, A. L., Neretti, N., Sedivy, J. M., & Kreiling, J. A. (2013). Transposable elements become active and mobile in the genomes of aging mammalian somatic tissues. *Aging (Albany NY), 5*, 867-883.

Elzein, S., & Goodyer, C. G. (2014). Regulation of human growth hormone receptor expression by micrornas. *Mol Endocrinol, 28*, 1448-1459. doi:10.1210/me.2014-1183

Friedlander, M. R., Chen, W., Adamidi, C., Maaskola, J., Einspanier, R., Knespel, S., & Rajewsky, N. (2008). Discovering micrornas from deep sequencing data using mirdeep. *Nat Biotechnol, 26*, 407-415. doi:10.1038/nbt1394

Friedman, R. C., Farh, K. K., Burge, C. B., & Bartel, D. P. (2009). Most mammalian mrnas are conserved targets of micrornas. *Genome Res, 19*, 92-105. doi:10.1101/gr.082701.108

Garcia, D. M., Baek, D., Shin, C., Bell, G. W., Grimson, A., & Bartel, D. P. (2011). Weak seed-pairing stability and high target-site abundance decrease the proficiency of lsy-6 and other micrornas. *Nat Struct Mol Biol, 18*, 1139-1146. doi:10.1038/nsmb.2115

Giardine, B., Riemer, C., Hardison, R. C., Burhans, R., Elnitski, L., Shah, P., . . . Nekrutenko, A. (2005). Galaxy: A platform for interactive large-scale genome analysis. *Genome Res, 15*, 1451-1455. doi:10.1101/gr.4086505

Goecks, J., Nekrutenko, A., & Taylor, J. (2010). Galaxy: A comprehensive approach for supporting accessible, reproducible, and transparent computational research in the life sciences. *Genome Biol, 11*, R86. doi:10.1186/gb-2010-11-8-r86

Grimson, A., Farh, K. K., Johnston, W. K., Garrett-Engele, P., Lim, L. P., & Bartel, D. P. (2007). Microrna targeting specificity in mammals: Determinants beyond seed pairing. *Mol Cell, 27*, 91-105. doi:10.1016/j.molcel.2007.06.017

Kent, W. J., Sugnet, C. W., Furey, T. S., Roskin, K. M., Pringle, T. H., Zahler, A. M., & Haussler, D. (2002). The human genome browser at ucsc. *Genome Res, 12*, 996-1006. doi:10.1101/gr.229102

Kozarewa, I., & Turner, D. J. (2011). Amplification-free library preparation for paired-end illumina sequencing. *Methods Mol Biol, 733*, 257-266. doi:10.1007/978-1-61779-089-8_18

Lewis, B. P., Burge, C. B., & Bartel, D. P. (2005). Conserved seed pairing, often flanked by adenosines, indicates that thousands of human genes are microrna targets. *Cell, 120*, 15-20. doi:10.1016/j.cell.2004.12.035

McCarthy, D. J., Chen, Y., & Smyth, G. K. (2012). Differential expression analysis of multifactor rna-seq experiments with respect to biological variation. *Nucleic Acids Res, 40*, 4288-4297. doi:10.1093/nar/gks042

Quail, M. A., Kozarewa, I., Smith, F., Scally, A., Stephens, P. J., Durbin, R., . . . Turner, D. J. (2008). A large genome center's improvements to the illumina sequencing system. *Nat Methods, 5*, 1005-1010. doi:10.1038/nmeth.1270

Robinson, M. D., McCarthy, D. J., & Smyth, G. K. (2010). Edger: A bioconductor package for differential expression analysis of digital gene expression data. *Bioinformatics, 26*, 139-140. doi:10.1093/bioinformatics/btp616

Trapnell, C., Pachter, L., & Salzberg, S. L. (2009). Tophat: Discovering splice junctions with rna-seq. *Bioinformatics, 25*, 1105-1111. doi:10.1093/bioinformatics/btp120

Vlachos, I. S., Kostoulas, N., Vergoulis, T., Georgakilas, G., Reczko, M., Maragkakis, M., . . . Hatzigeorgiou, A. G. (2012). Diana mirpath v.2.0: Investigating the combinatorial effect of micrornas in pathways. *Nucleic Acids Res, 40*, W498-504. doi:10.1093/nar/gks494
